# A novel *KIDINS220* mutation associated with hereditary spastic paraplegia accompanied by severe peripheral neuropathy

**DOI:** 10.3389/fnins.2025.1684980

**Published:** 2025-10-14

**Authors:** Xujun Chu, Jin Xu, Yilei Zheng, Xiaoyu Liu, Yun Yuan, Rui Wu

**Affiliations:** ^1^Department of Neurology, Shandong Provincial Hospital Affiliated to Shandong First Medical University, Jinan, China; ^2^Lab of Electron Microscopy, Center of Ultrastructural Pathology, Peking University First Hospital, Beijing, China; ^3^Department of Neurology, Peking University First Hospital, Beijing, China

**Keywords:** *KIDINS220*, hereditary spastic paraplegia, peripheral neuropathy, nerve biopsy, genotype–phenotype correlation

## Abstract

**Objectives:**

Mutations in *KIDINS220* are known to cause hereditary spastic paraplegia (HSP) and SINO syndrome. However, the phenotypic and genotypic spectrum of *KIDINS220*-related disorders remains incompletely understood. Herein, we describe the clinical, electrophysiological, histopathological, and genetic features of a novel KIDINS220 sterile alpha motif (SAM) -like domain mutation identified in a Chinese family with HSP accompanied by severe peripheral neuropathy (PN).

**Methods:**

Clinical data, electrophysiological characteristics, and sural nerve histopathology were analyzed in a 19-year-old Chinese male. Genetic testing was performed in his family by using whole-exome sequencing, mitochondrial genome testing, and Sanger validation. A comprehensive literature review was conducted to analyze the phenotypic and genetic data of previously reported cases with *KIDINS220* variants up to July 2025.

**Results:**

The proband exhibited classical signs of autosomal dominant HSP accompanied by severe multifocal sensory-motor PN. The spinal cord MRI showed mild spinal cord thinning, while the brain MRI and nerve ultrasound examinations were normal. Electrophysiological study revealed absent sensory nerve responses and globally reduced motor conduction velocities. Sural nerve biopsy confirmed significantly reduced nerve fiber density, myelin defects, axonal degeneration, and mitochondrial abnormalities. A heterozygous *KIDINS220* c.3668A > G (p. Glu1223Gly) mutation, located within the SAM domain, was identified in both the proband and his mother. A total of 42 cases from 11 cohorts were reviewed.

**Conclusion:**

We suggest that patients with KIDINS220 SAM domain mutation may present with HSP accompanied by severe, mixed axonal and demyelinating PN, expanding the existing spectrum of the clinical phenotypes and pathogenic variants of *KIDINS220*.

## Introduction

Hereditary spastic paraplegia (HSP) is a heterogeneous group of inherited neurodegenerative disorders, with an estimated prevalence of fewer than 10 cases per 100,000 individuals (Fink, 2023). The primary pathogenesis of HSP is axonal degeneration of the corticospinal tracts, resulting in progressive decline in motor function (Sardina et al., 2024). Affected individuals typically present with lower-limb spasticity, weakness, and hyperreflexia (Wiseman et al., 2024; Yilmaz et al., 2025; Zhao et al., 2019). As the disease progresses, gait abnormalities and postural instability frequently develop, further affecting mobility and quality of life (Koch et al., 2025; Regensburger et al., 2022). Even though patients have above shared features, HSP demonstrates substantial clinical heterogeneity, and is classified into pure and complex forms based on the presence or absence of additional neurological or non-neurological deficits. The pure form involves isolated spastic paraplegia, whereas the complex form is accompanied by intellectual disability, ataxia, epilepsy, axonal or demyelinating peripheral neuropathy (PN), and craniofacial dysmorphism (Diarra et al., 2024; Panza et al., 2022). To date, more than 90 subtypes of HSP have been described, each due to mutations in a different gene, suggesting the extensive genetic heterogeneity of HSP (Koch et al., 2025). These genes encode proteins playing critical roles in a variety of cellular processes involved in maintaining neuronal integrity and function (Garg et al., 2024).

Kinase-D interacting substrate of 220 kDa gene (*KIDINS220*: MIM*615759) has been recently identified as a causative gene for a complex form of HSP termed SINO syndrome, characterized by spastic paraplegia, intellectual disability, nystagmus, and obesity (Josifova et al., 2016). The protein encoded by *KIDINS220* is ubiquitously expressed in the central and peripheral nervous systems, where it functions as a pivotal regulator of neurodevelopment. Specifically, it participates in neurotrophin-mediated signaling pathways, including those of nerve growth factor and brain-derived neurotrophic factor, and is essential for neuronal survival, axonal guidance, and synaptic plasticity (Josifova et al., 2016; Neubrand et al., 2012; Scholz-Starke and Cesca, 2016). Previous studies have suggested that the spectrum of *KIDINS220* mutations and their associated disorders may reflect a potential genotype–phenotype correlation, influenced by inheritance pattern, molecular consequence, affected protein domains, and modifier genes. However, the currently available literature on *KIDINS220* mutations is very limited. Additional genotypic and phenotypic variants remain to be further discovered.

We herein describe a patient harboring a novel KIDINS220 sterile alpha motif-like (SAM) domain variant c.3668A > G (p. Glu1223Gly) who presented with HSP accompanied by severe, mixed axonal and demyelinating PN. Histopathological examination of sural nerve was performed to investigate potential underlying pathogenesis. To the best of our knowledge, there is no documented evidence suggesting that *KIDINS220* mutations cause severe PN. These findings suggest that the variant may represent a novel genotype–phenotype correlation, extending the current understanding of the mutational and phenotypic spectrum of *KIDINS220*-related disorders.

## Materials and methods

### Subjects

A mainland Chinese family with clinically suspected HSP was enrolled in this study, which was approved by the Ethics Committee of the Shandong Provincial Hospital, Jinan, Shandong, China, and was conducted in accordance with the Declaration of Helsinki. Written informed consents were obtained from the proband and his family members.

**FIGURE 1:**
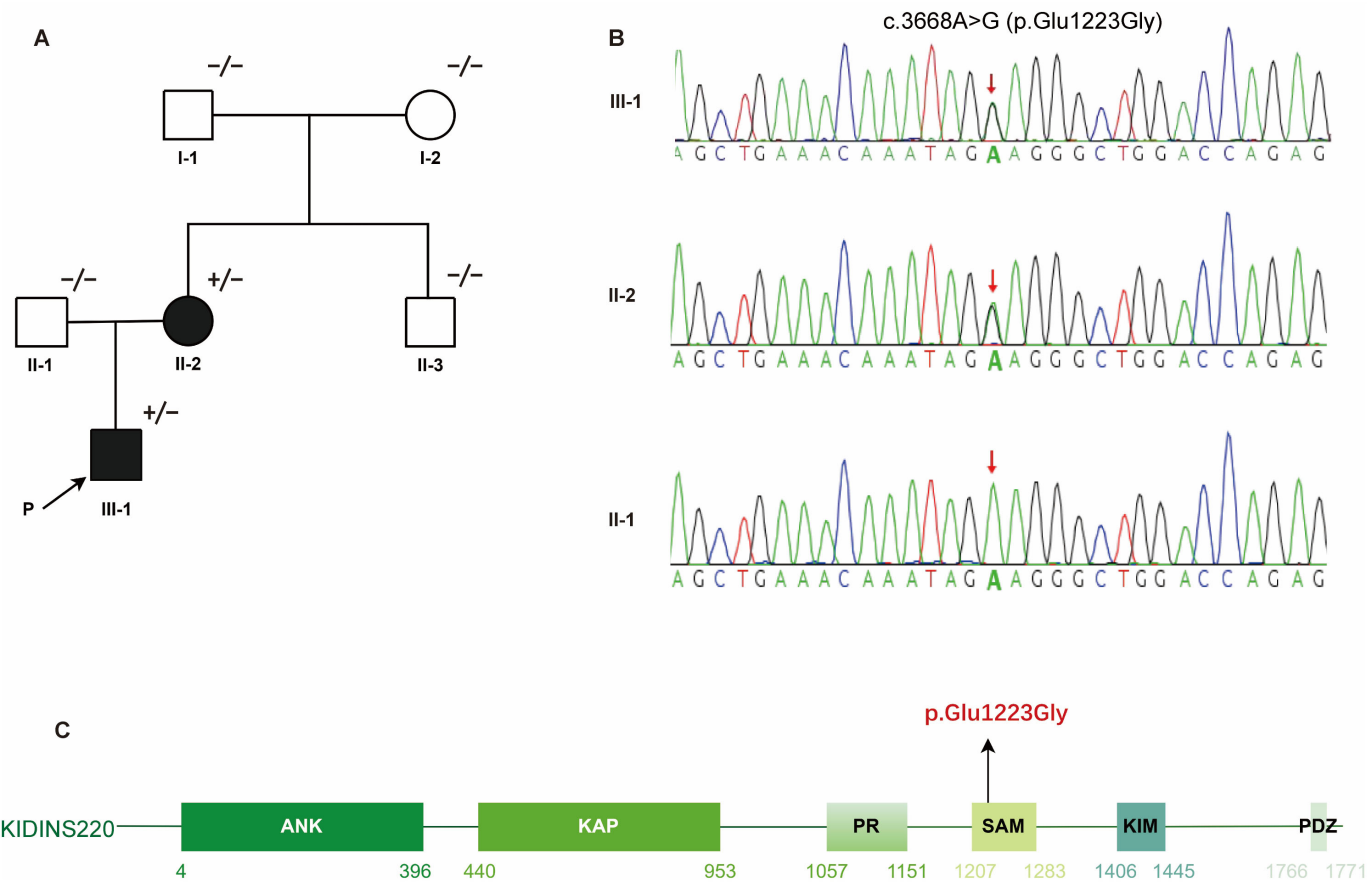
Identification of a heterozygous *KIDINS220* c.3668A > G (p. Glu1223Gly) mutation in a HSP family. **(A)** Pedigree of the affected family carrying the *KIDINS220* mutation. Symbols with “+/−” indicate mutation carriers; symbols with “−/−” indicate non-mutation carriers. Filled or empty symbols represent individuals with or without symptoms. The proband is indicated by the arrow. **(B)** Sanger sequencing validated the heterozygous c.3668A > G (p. Glu1223Gly) variant detected via WES in member III-1 and II-2. The red arrow indicates the mutation site. **(C)** Schematic representation of the six predicted functional domains of *KIDINS220*: ankyrin repeats domain (ANK), after Kidins220/ARMS and PifA-NTPase domain (KAP); proline-rich domain (PR), sterile alpha motif-like domain (SAM); kinesin1-interacting motif domain (KIM), and PSD-95, Dlg, and ZO-1 proteins-binding motif domain (PDZ). The disease-causing mutation c.3668A > G (p.Glu1223Gly) identified in this study is highlighted in red. HSP, hereditary spastic paraplegia; WES, whole-exome sequencing.

The proband (III-1, Figure 1A) was a 19-year-old male born to non-consanguineous parents. He presented with bilateral lower limb weakness and stiffness at the age of 10. Symptoms progressed slowly leading to an unsteady gait. He also complained slight numbness in both hands and feet. He denied history of intellectual disability, epilepsy, visual or auditory impairment, or obesity. Physical examination revealed that his body mass index was 21 kg/m^2^ and no deformities were noted. His cognitive score was 29/30 measured using the Montreal Cognitive Assessment. Cranial nerve functions were normal. Muscle weakness was predominant in the proximal lower limbs with hip flexion grade 4/5 (Medical Research Council scale). Muscle strength was grade 5/5 in other muscle groups with increased muscle tone in all four limbs. Sensory examination revealed decreased pinprick, vibration, and temperature sensation distal to the elbows and below the knees. Hyperreflexia, bilateral pyramidal signs, and ankle clonus were present. No nystagmus, dysarthria, or ataxia was observed.

In this family, the proband had an affected mother (II-2, Figure 1A) with similar symptoms. However, she reported lifelong lower limb weakness and gait abnormalities without notable progression. She denied numbness in the limbs. On physical examination, her proximal lower limb strength was 4/5 and other muscle groups was 5/5. Increased muscle tone, hyperreflexia, and bilateral positive pyramidal signs were present, while sensory and cerebellar examinations were unremarkable.

Laboratory testing of the proband revealed normal serum creatine kinase, blood glucose and homocysteine levels. No abnormalities were detected in the electrocardiogram and echocardiography.

### Nerve conduction study, nerve ultrasonography, neuroimaging, and nerve biopsy

Nerve conduction study (NCS) and nerve ultrasonography were performed in the proband and her affected mother. Neuroimaging examinations in the proband included brain, entire length of spinal cord, and lower limb muscle magnetic resonance imaging (MRI). A right sural nerve biopsy was further performed in the proband. The nerve specimens were processed using standard methods as follows: a larger piece of the specimen was fixed in 4% formalin, embedded in paraffin, and stained with hematoxylin-eosin, Congo Red, and Luxol Fast Blue. Immunohistochemistry staining includes myelin basic protein and neurofilament. Another part of the nerve sample was fixed in 3% glutaraldehyde and postfixed in 1% osmium tetroxide. Semithin sections were stained with Toluidine Blue for observation under light microscopy. Ultrathin sections were contrasted with uranyl acetate and lead citrate and later examined under electron microscopy. The nerve fiber density and diameter were calculated using NIS-Elements BR 3.2 program.

### Genetic analysis

Genomic DNA was extracted from whole peripheral blood samples from the proband and his family members. The proband was sequenced using whole exome sequencing (WES), and mitochondrial genome testing. The pathogenicity of candidate variants was predicted by using multiple *in silico* algorithms such as mutation taster,^1^ FATHMM,^2^ SIFT,^3^ provean,^4^ and was classified according to American College of Medical Genetics and Genomics (ACMG) guidelines. Sanger sequencing was used to validate the filtered variants in the family members of the patient. Finally, the variants were selected due to their relationship with the disease, pattern of segregation with the disease, pattern of inheritance, allele frequency in controls, and predicted pathogenicity.

### Literature review

The English-language literature related to the *KIDINS220* was searched in PubMed up to July 2025. The search terms included “*KIDINS220*” and/or “SAM domain,” “hereditary spastic paraplegia,” “peripheral neuropathy,” “nerve biopsy,” “genotype–phenotype correlation.” Cases without detailed clinical information were excluded.

## Results

### Nerve electrophysiological findings

Table 1 summarizes the nerve electrophysiological data. The NCS in the proband showed diffusely slowed motor conduction velocities and reduced compound muscle action potential amplitudes (CMAPs). All sensory nerve action potentials (SNAPs) were not recordable, indicating that sensory nerve involvement was severer than motor nerves. In contrast, the NCS in the proband’s mother revealed relatively mildly slowed conduction velocities limited to sensory nerves.

**TABLE 1:** Nerve conduction studies in patients with c.3668A > G in the *KIDINS220* gene.

Patient	Motor nerves						Sensory nerves	
	Median		Peroneal		Median		Superficial peroneal	
	CMAP (mV)	MNCV (m/s)	CMAP (mV)	MNCV (m/s)	SNAP (μV)	SNCV (m/s)	SNAP (μV)	SNCV (m/s)
Proband (III-1)	5.9	**43.5**	**0.3**	**31.9**	**–**	**–**	**–**	**–**
Mother (II-2)	8.8	51.9	1.6	46.7	19.3	**43.8**	5.2	**38.1**

CMAP, compound muscle action potential; MNCV, motor nerve conduction velocity; SNAP, sensory nerve action potential; SNCV, sensory nerve conduction velocity; “–”, not recordable. The bolded values indicate the presence of anomalies.

### Nerve ultrasonography and neuroimaging findings

Peripheral nerve ultrasound revealed no abnormalities in the nerve cross-sectional area or echogenicity. In the proband, slight atrophy of thoracal spinal cord was seen on MRI, while brain and muscle MRI were unremarkable.

### Nerve pathology

**FIGURE 2:**
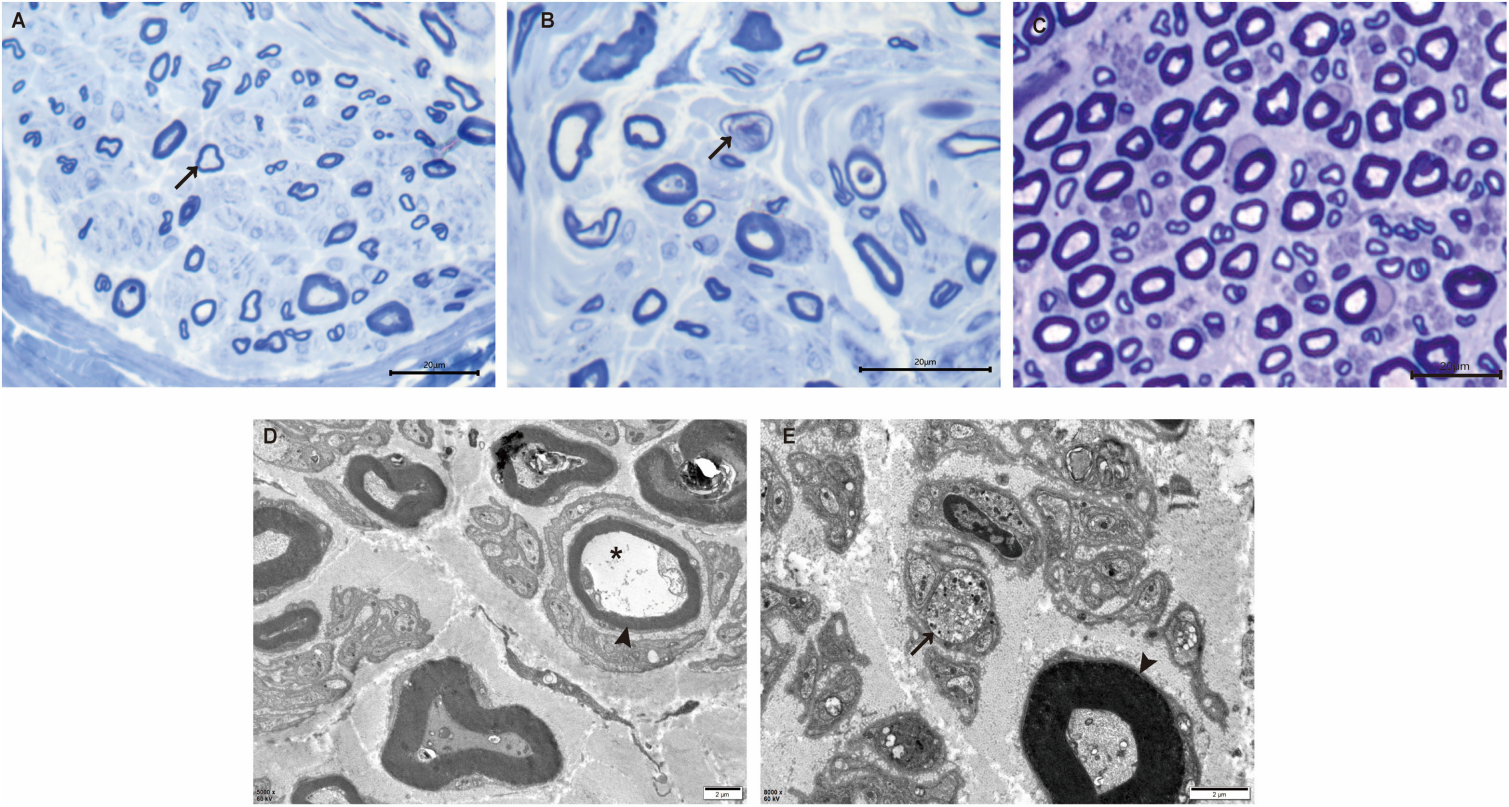
Nerve pathology of the proband. **(A)** Toluidine-stained semithin section of the proband shows loss of myelinated nerve fibers and thinly myelinated fibers (arrow). **(B)** Semithin section of the proband shows axonal degeneration (arrow). **(C)** Semithin section of an age-matched control. **(D)** Electron microscopy reveals the presence of thinly myelinated fibers (arrowhead) and axonal degeneration in myelinated fibers (*). **(E)** Axonal degeneration, abnormal aggregation and structural disruption of mitochondria are observed in unmyelinated fibers (arrow). Thickened myelinated fibers (arrowhead) are also present. Scale bars: 20 μm **(A–C)**, 2 μm **(D,E)**.

**FIGURE 3:**
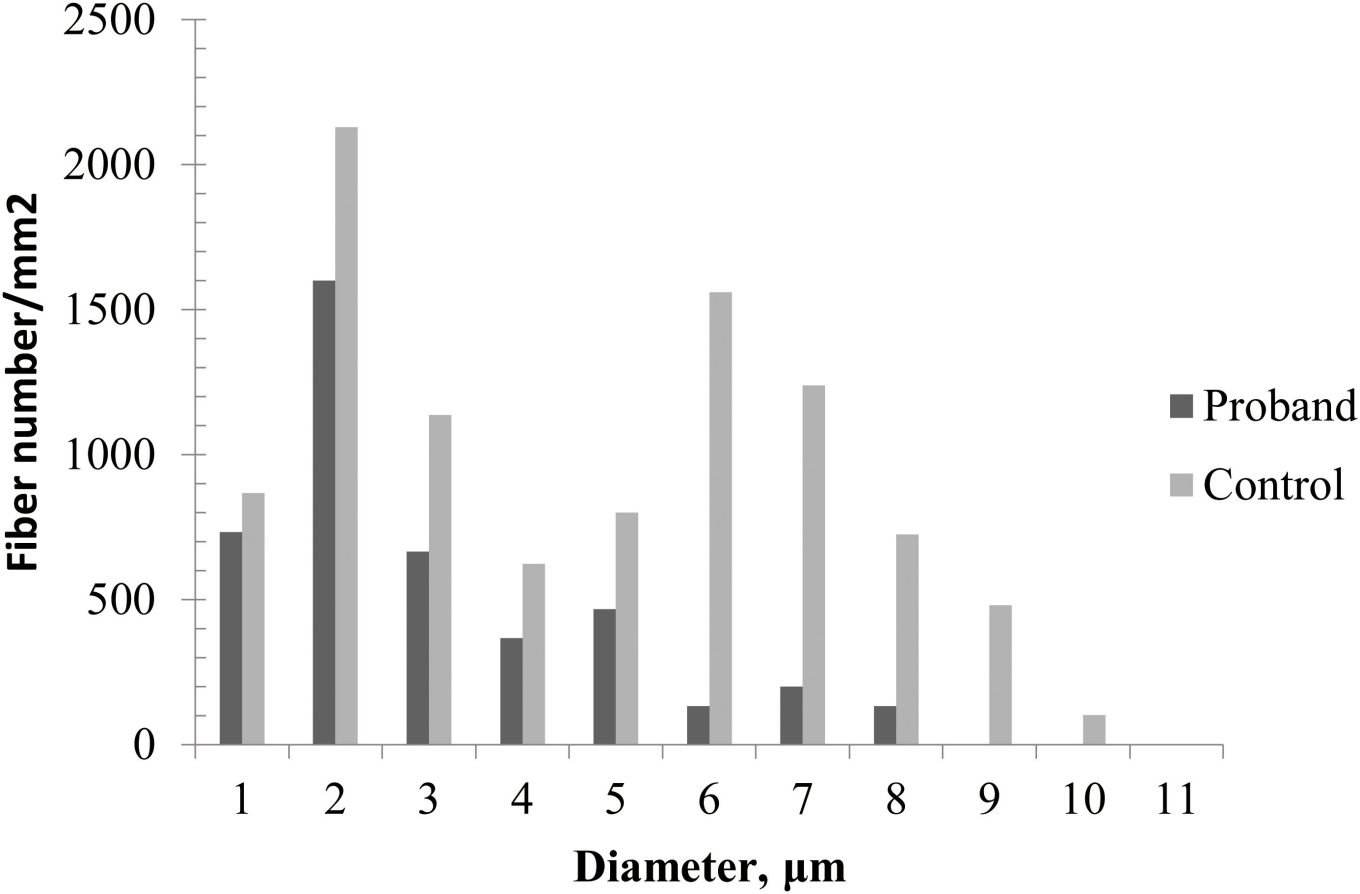
Quantitative analysis of sural biopsy specimens. The histogram shows the unimodal distribution pattern of myelinated fibers with loss of large myelinated fibers and low fiber density in the proband compared with an age-matched control.

Sural nerve biopsy demonstrated a markedly decreased density of myelinated fibers (Figure 2A) compared with an age-matched control (Figure 2C). Thinly myelinated fibers (Figure 2A) and axonal degeneration (Figure 2B) were seen without evidence of onion-bulb formation and axonal regeneration clusters. Electron microscopy (Figures 2D, E) further confirmed axonal degeneration in both myelinated and unmyelinated fibers. Myelin abnormalities, including both thinning and thickening, were also observed. Abnormal aggregation and structural disruption of mitochondria were noted. Quantitative analysis reveals that the mean density of myelinated fiber was 4900/mm^2^ (8112/mm^2^ in the control). According to published reference data, the average number of myelinated nerve fiber in young adults is approximately 7,000–10,000 fibers/mm^2^ of endoneurial area (Amato and De Girolami, 2023). The histogram of the presumptive myelinated fibers was skewed to the left, indicating a loss of large myelinated fibers (Figure 3).

### Genetic analysis

WES identified a heterozygous KIDINS220 c.3668A > G (p. Glu1223Gly) variant in the proband as well as in his mother (Figure 1B). This variant was not detected in the other asymptomatic family members. Mutations in genes related to Charcot-Marie-Tooth disease (PMP22, MPZ, MFN2, GJB1, etc.) and in mitochondrial genome were not detected. This variant is located in the functional SAM domain of KIDINS220 (Figure 1C), which is a conserved residue. Neither variant is listed in dbSNP, the 1000 Genomes Project, or in the 20,000 Chinese controls. Results of *in silico* analysis for variant pathogenicity were predicted to be “damaging/pathogenic.” This variant was classified as “likely pathogenic” based on the ACMG guidelines.

### Literature review

A total of 42 cases from 11 studies harboring pathogenic *KIDINS220* variants were reviewed from diverse ethnic backgrounds (mainly from China, Europe, USA, and Egypt). Of the 33 surviving cases (79%), 30 cases (91%) onset before the age of 10. Eighteen cases presented with SINO syndrome, including 18 (100%) with intellectual disability, 17 (94%) with obesity and 16 (89%) with nystagmus. The other 15 cases manifested as pure HSP. Additionally, some cases exhibited rare manifestations such as talipes equinovarus, impaired vision, and dysarthria. PN was not reported in any of the previously published *KIDINS220* cohorts. Among these surviving cases, the vast majority (97%) exhibited dominant inheritance. Only one case had a recessive inheritance pattern; however, abnormalities occurred *in utero* and the symptoms were severe and rapidly progressed after birth. All the other cases with recessive inheritance died during the fetal period. Prenatal examinations of these patients revealed dilated cerebral ventricles (100%), limb contractures (89%), cerebellar hypoplasia (33%), and other neurodevelopmental anomalies such as agenesis of corpus callosum, brachycephaly, micrognathia. Therefore, it can be seen that the recessive inheritance pattern of *KIDINS220* mutations is more disastrous for the patients, highlighting a potential severity threshold associated with mutation type and zygosity.

Genetic analysis revealed significant allelic heterogeneity, with at least 20 distinct variants reported, including missense (e.g., p.E1223G, p.N728I), nonsense (e.g., p.W1350X, p.Q1393*), as well as frameshift and splice site mutations (e.g., p. L1507Ffs*4, IVS27-12A > G). Most of these mutations are located downstream of the SAM domain.

## Discussion

This study reported a patient harboring the novel *KIDINS220* c.3668A > G (p. Glu1223Gly) variant who exhibited autosomal dominant HSP accompanied by severe, multifocal PN, but without features of SINO syndrome. This variant was not detected from large population databases such as gnomAD. In combination with the pathogenic prediction by using multiple *in silico* algorithms and its segregation with disease in the family, its pathogenicity was further confirmed. To the best of our knowledge, this is the first report to associate this variant with a specific phenotype, thereby expanding the mutational and phenotypic spectrum of *KIDINS220*-related disorders.

In this study, we reviewed 42 cases from 11 cohorts harboring pathogenic *KIDINS220* variants. In Josifova et al. (2016), heterozygous mutations in *KIDINS220* were discovered to be pathogenic and can cause SINO syndrome. Yang et al. (2018) reported the first Chinese case harboring a heterozygous variant presenting with HSP, obesity, and developmental delay. Subsequent studies in Chinese cohorts identified additional cases carrying heterozygous *KIDINS220* mutations, with a subset exhibiting as pure form (Zhao et al., 2019). The first surviving patient with autosomal recessive (AR) *KIDINS220* mutations was reported in 2022, exhibiting a severer pre-and postnatal presentation, including ventriculomegaly, limb contractures, and features of SINO syndrome, in contrast with prior cases of intrauterine lethality in homozygous individuals (Wiseman et al., 2024). Additional reports further confirmed that AR *KIDINS220* variants are commonly associated with a more severe phenotype compared to their dominant counterparts (Yang et al., 2024). These cases exhibited significant clinical heterogeneity, with phenotypes ranging from intrauterine death and neurodevelopmental abnormalities (El-Dessouky et al., 2020), or progressive cognitive decline after birth (Brady et al., 2022), to pure HSP without syndromic features (Zhao et al., 2019). While many previously reported patients presented with SINO syndrome features, which includes spastic paraplegia, intellectual disability, nystagmus, and obesity, our patient showed only spastic paraplegia without other syndromic manifestations. Furthermore, none of the previously described cases, including the recent comprehensive analysis by Alstrup et al. (2024), reported PN of the severity observed in our patient. Therefore, in this cohort we described the detailed electrophysiological and pathological changes of the PN, in order to further expand the clinical spectrum and deepen the understanding of the mechanisms of neurological damage associated this *KIDINS220* mutations.

HSP was traditionally considered a central nervous system–restricted distal motor axonopathy. However, accumulating evidence indicates that some HSP subtypes are accompanied by other neurological comorbidities. PN is one of the key manifestations of complex HSP, often occurring alongside cognitive impairment, cerebellar ataxia, epilepsy, and visual disturbances. For instance, PN has been documented in SPG31 due to *REEP1* mutations, with some cases presenting with carpal tunnel syndrome (Toft et al., 2019). A Chinese cohort further demonstrated that PN-confirmed by sensory loss and/or electrophysiological abnormalities-was present in 2 of 5 SPG10 patients (Cao et al., 2024). Other HSP subtypes associated with PN may include SPG2, SPG3A, SPG5, SPG6, SPG7, SPG10, SPG25, SPG27, SPG30, SPG31, SPG55, SPG56, SPOAN syndrome, and mutations in the mitochondrial *ATP6* gene. Notably, the peripheral nerve involvement was generally mild in previous cohorts. In contrast, the present case of *KIDINS220*-related HSP demonstrated significantly more severe peripheral nerve impairment, as evidenced by clinical presentation and electrophysiological assessments. This resulted in a prior misdiagnosis of Charcot-Marie-Tooth disease in the proband before the current evaluation. Such heterogeneity of PN among HSP subtypes may reflect the diverse molecular functions of the underlying mutant proteins.

Sural nerve biopsy in our patient revealed chronic mixed axonal and demyelinating PN, providing the first pathological evidence implicating *KIDINS220* mutations in the pathogenesis of PN. In contrast to the limited literature describing peripheral nerve histopathology in HSP, which showed only mild reduction of fiber density, our patient exhibited more severe and complex pathological changes, consistent with the electrophysical findings. In addition, quantitative analysis of the sural nerve biopsy revealed that fiber loss predominantly affected large-diameter myelinated fibers. Although small fibers were also reduced, they appeared relatively preserved. This selective pattern of fiber involvement may explain the discrepancy between absent SNAPs on electrophysiological testing, which primarily reflect the function of large myelinated fibers, and the patient’s partially preserved sensory perception, which is compensated for by small-diameter fibers.

The concurrent involvement of the corticospinal tract and peripheral nerves in our patient suggests that KIDINS220 may function at multiple levels of the nervous system. It is known that KIDINS220 interacts with microtubule-associated proteins, including MAP1B and tubulin. Thus, mutant KIDINS220 may disturb microtubule stability in neurons, thereby impairing axonal transport in the corticospinal tract and peripheral nerves, and ultimately contributing to the development of spastic paraplegia and PN (Bonati et al., 2024). Besides, variant identified in this cohort is located within the SAM domain, and to the best of our knowledge, represents the first reported *KIDINS220* mutation affecting this structural domain. Although the precise role of the SAM domain in KIDINS220 remains incompletely understood, it is known to mediate inter- and intramolecular interactions, such as protein oligomerization or act as scaffolds in signaling cascades (Neubrand et al., 2012). Impairment of this domain may disrupt electrostatic interaction, protein-protein docking, or domain stability that regulate neuronal survival and apoptosis, potentially leading to distal axonal degeneration—also known as “dying-back” neuropathy—manifesting clinically as PN (Scholz-Starke et al., 2012). As shown in the proband’s sural nerve histopathology, the presence of axonal degeneration and absence of regeneration clusters reflect the pathogenesis. Moreover, *KIDINS220* is implicated in modulating synaptic plasticity, particularly within sensory neuronal circuits. Mutations may therefore affect the development and integrity of the sensory nervous system, resulting in severely impaired sensory conduction shown in the present case. The multifunctionality and structural complexity of *KIDINS220* may underlie the phenotypic heterogeneity associated with its mutations, suggesting that different mutation sites may result in either pure or complex HSP subtypes (Yang et al., 2024).

It is noteworthy that the patient’s mother, who carried the same variant, exhibited a milder phenotype with non-progressive symptoms. Similar variable expressivity can also be seen in the reviewed cohorts (Zhang et al., 2021). This phenomenon is potentially influenced by epigenetic modifiers, age, sex-specific regulatory factors, or gene dosage effects (Albini et al., 2024). Longitudinal studies are warranted to investigate the modulators of phenotypic variability in *KIDINS220*-related disorders.

This study is limited by the lack of functional validation of the variant *in vitro* or *in vivo*. To date, no experimental studies have assessed the functional consequences of the c.3668A > G variant, and its specific impact on the structure or biological role of the SAM domain has not yet been elucidated. In future work, we plan to conduct *in vitro* studies using patient derived induced pluripotent stem cell models and neuronal cell lines expressing the mutant KIDINS220 to identify the pathogenic mechanisms of SAM domain dysfunction. These studies will evaluate the variant’s impact on protein stability, subcellular localization, and downstream molecular signaling pathways. We also aim to develop animal models to investigate the effects on both central and peripheral nervous system development. Furthermore, the patient exhibited reduced temperature sensation, indicating broader involvement of nociceptive pathways. However, other aspects of nociception, such as burning pain, cold pain, and pressure pain, were not systematically tested. Given the known role of KIDINS220 in nociceptive pathways (Sanchez-Sanchez et al., 2023), further studies including quantitative sensory testing are necessary to comprehensively evaluate nociceptive function in patients with similar variants.

In conclusion, the present case of HSP accompanied by severe, mixed axonal and demyelinating PN provides novel insight into the pathogenesis of *KIDINS220*-related disorders. It expands the existing spectrum of both the clinical phenotypes and pathogenic variants of *KIDINS220*. It also highlights that *KIDINS220* should be considered in the genetic workup for HSP with marked PN.

## Data Availability

The original contributions presented in this study are included in this article/supplementary material, further inquiries can be directed to the corresponding author.

## Footnotes

1
http://www.mutationtaster.org/

2
https://fathmm.biocompute.org.uk/

3
http://sift.jcvi.org/

4
http://provean.jcvi.org/
